# Validation of a Feed Protocol in a Mouse Model That Mimics Marasmic Malnutrition

**DOI:** 10.3389/fvets.2021.757136

**Published:** 2021-11-29

**Authors:** Taiana Ferreira-Paes, Paula Seixas-Costa, Elmo Eduardo Almeida-Amaral

**Affiliations:** Laboratório de Bioquímica de Tripanosomatídeos, Instituto Oswaldo Cruz/Fundação Oswaldo Cruz, Rio de Janeiro, Brazil

**Keywords:** malnutrition, refeeding, mice, phenotypic markers, marasmic malnutrition

## Abstract

Host nutritional status directly interferes with immunity and/or susceptibility to infectious diseases. To understand the mechanisms behind this relationship, the use of animal models and feeding protocols is necessary. In the literature, studies reporting marasmic malnutrition in mice are not common. In this context, the objective of this study was to validate a feed methodology that mimics marasmic malnutrition, examining the nutritional, biochemical, and hematological status in BALB/c mice. Weaned BALB/c mice were or were not fed a Restricted diet (36.26% carbohydrate, 8.79% protein, 4.95% fat, and 7.62 kJ/100 g). Some malnourished mice underwent a refed process with a Control diet (65.93% carbohydrate, 24.18% protein, 9.89% fat, and 15.24 kJ/100 g). The nutritional status of the mice was evaluated through phenotypic markers and hematological and biochemical parameters. Our results showed that the Restricted diet was able to induce mild malnutrition in mice, resulting in mouse weight loss of 12%, which could be reversed after refeeding. Malnourished mice demonstrated slow body growth and low body mass index (BMI) values. Malnourished mice also showed physical and behavioral changes, a reduction of 47.5% in leukocyte counts and a 2-fold increase in cholesterol levels. In conclusion, our feeding protocol was able to generate mild malnutrition and cause changes in the nutritional status of mice that could be similar to those observed in marasmic malnutrition.

## Introduction

Malnutrition is a nutritional deficiency caused by inadequate intake of macronutrients (proteins, carbohydrates, and lipids) and micronutrients (vitamins and minerals) essential for growth and physical and mental development (1–4). According to a report published by the Food and Agriculture Organization of the United Nations (FAO), it is estimated that 728 million people worldwide were malnourished in 2020. In Latin America and the Caribbean, malnutrition rates have increased in recent years, mainly in South America, where 33.7 million people are malnourished (5).

Protein-energy malnutrition (PEM) is the most prevalent type of malnutrition in the world and is divided into three forms: kwashiorkor, marasmus, and an intermediate stage named marasmic-kwashiorkor. This form presents a set of clinical features that vary according to the degree of nutritional deficiency. Furthermore, the etiology of these malnutrition forms is not entirely clear (6–8). It commonly describes kwashiorkor as edematous malnutrition that generates clinical features such as hair changes, skin lesions, hepatic abnormalities (hepatomegaly and fatty infiltrations) and the cardinal signal, edema (6–11). Marasmus is frequently described as non-edematous malnutrition characterized by severe weight loss, muscle atrophy, absence of subcutaneous fat and edema, and low weight-for-height (4, 6, 8–13). Marasmic-kwashiorkor is a combination of clinical features of both malnutrition forms, including the presence of edema, subcutaneous fat loss, and muscle wasting (8, 10, 13). Although the etiology is unclear, kwashiorkor is often associated with protein-deficient diets, and marasmus is associated with calorie-restricted diets that overcome protein deficiency. Marasmic-kwashiorkor can be associated with diets deficient in both calories and proteins. Low consumption of micronutrients also contributes to malnutrition forms (6, 8, 12).

Previous studies have described that malnutrition, mainly PEM, is closely associated with infections since it causes human immunodeficiency worldwide (14–16). Frequently, millions of malnourished people, mostly children under the age of 5 years old, die from infections (17). Low nutrient intake affects innate and acquired immunity, leading to the host's inability to respond to infection and impairing cell and organ function (18, 19).

Although the host's nutritional status affects immunity and/or susceptibility to infectious diseases, infections may also impair nutrient uptake by the host, which contributes to malnutrition (20–23). Due to this intrinsic relationship and the high degree of mortality caused by malnutrition, it is important to understand the contribution of malnutrition to worsening diseases and the role of nutrition in preventing them (19, 24). In the literature, it is very common to find works portraying the relationship of protein malnutrition with different infectious diseases, such as leishmaniases (14, 25, 26), intestinal parasite infection (27, 28) and Zika (16). Other studies have demonstrated the effects of a lack of micronutrients on susceptibility to diseases (29, 30). However, few studies use methodologies that mimic marasmus PEM in a mouse model or that analyze the nutritional status of mice beyond body weight. Therefore, the aim of this study was to validate a methodology to mimic marasmic malnutrition in BALB/c mice and validate this nutritional status of mice through phenotypic markers as well as hematological and biochemical parameters. This protocol could be used to experimentally investigate the relationship between marasmic malnutrition and impaired immunity and/or susceptibility to infectious diseases.

## Materials and Methods

### Mice

Weanling female BALB/c mice (3 weeks old) were obtained from Instituto de Ciência e Tecnologia em Biomodelos (ICTB—FIOCRUZ, Rio de Janeiro, Brazil). All procedures involving animals were reviewed and approved by the Ethics Committee on the Use of Animals at Instituto Oswaldo Cruz (license L-011/2017).

### Diets

The Control diet was composed of 24.18% crude protein, 65.93% carbohydrate, and 9.89% ethereal extract (fat), providing 15.24 kJ/100 g. The Restricted diet was a hypocaloric/hypoprotein/hypolipidic diet composed of 8.79% crude protein, 36.26% carbohydrate and 4.95% ethereal extract (fat), providing 7.62 kJ/100 g. Both diets were completed with fibers and contained all essential micronutrients for mouse survival. Control diet composition (macronutrients) was based on NUVILAB CR-1 ration (Nuvital Nutrientes S/A, Colombo, PR, Brazil), that is used in animal facilities. Restricted diet composition was based on our need to seek a diet with total macronutrient restriction, keeping within the minimum values for the mice nutrition. The objective was to use a diet capable of mimicking marasmatic malnutrition. Experimental diets were formulated by PragSoluções (PragSoluções Biociências, Jaú, SP, Brazil), and a detailed description of their composition is shown in Table 1.

**Table 1:** Composition of experimental diets.

**Composition**	**Control diet**			**Restricted diet**		
	**Amount (g)**	**Calories (kJ)**	**Calories (%)**	**Amount (g)**	**Calories (kJ)**	**Calories (%)**
Mineral matter	8	0	0	7.7	0	0
Crude protein	22	3.68	24.18	8	1.34	8.79
Ethereal extract (fat)	4	1.51	9.89	2	0.75	4.95
Crude fiber	6	0	0	49.3	0	0
Carbohydrate	60	10.05	65.93	33	5.53	36.26
Total	100	15.24	100	100	7.62	50

*Basic components: ground corn, soybean meal, wheat bran and/or Promil and a mixture of minerals, vitamins and amino acids*.

### Feeding Protocol and Experimental Procedure

**Figure 1:**
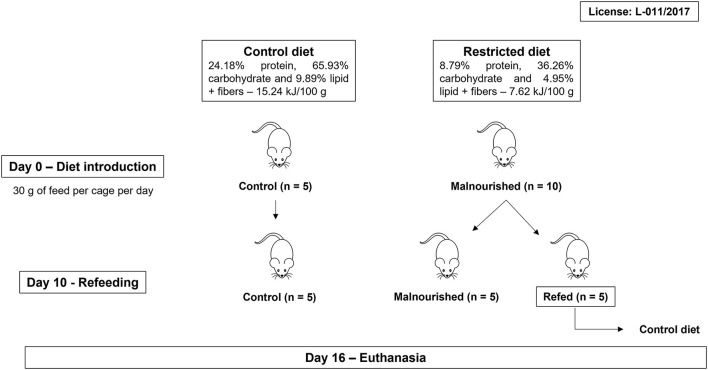
Workflow of feeding protocol. BALB/c mice were randomly divided into two groups (control and malnourished groups). Mice in the control group received the Control diet, and mice in the malnourished group received the Restricted diet. On day 10, the malnourished group was randomly divided again, forming two groups: the malnourished group and the refed group. The malnourished group continuously received the Restricted diet, while the refed group received the Control diet. After 16 days, the mice were euthanized. Three independent experiments were performed with five animals per group, totalizing 15 animals/group.

BALB/c mice received the Control diet (30 g/cage/day) for 1 week to acclimate. After that, the mice were randomly divided into two groups: control and malnourished. Mice in the control group received 30 g/cage/day Control diet, and mice in the malnourished group received 30 g/cage/day Restricted diet. After day 10, the malnourished group was randomly divided into two other groups: the malnourished group and the refed group. The malnourished group continuously received 30 g/cage/day of Restricted diet until day 16, while the refed group started receiving 30 g/cage/day of Control diet until day 16 (Figure 1). At day 0 (diet introduction), all animal group started with an average weight of 19 g. In each experiment, mice were housed at five per cage and had free access to water. The experiments were performed 3 times, totaling 15 animals/group. The rations per cage that were not consumed were weighed daily and completed for 30 g. In addition, the feed, water and calorie consumption were calculated daily: consumption was calculated by subtracting the remaining feed from the amount of feed provided the previous day.

### Phenotypic Markers

To analyze nutritional status, all mice were weighed daily on a digital scale to determine their body weight. In each group, mice started with an average weight of 19 g. At the end of the experiment (day 16), the weight gain percentage was analyzed by dividing the final weight (day 16) by the initial weight (day 0) of the mice (control and malnourished mice). In contrast, for the refed mice, the weight on the last day before refeeding (day 10) was divided by the initial weight (day 0), and the weight immediately before refeeding (day 11) was divided by the final weight (day 16). Body length (nose-to-anus length) was measured with a measuring tape at 0, 3, 10, and 16 days of the experiment. Body weight and body length were used to determine the body mass index (BMI) with the following equation:


$$\begin{array}{l} Ȃ\text{ Body mass index }=\;\frac{\text{body weight }\left(\text{g}\right)}{\text{nose}-\text{to}-\text{anus lengt}{\text{h}}^{2}\;\left(\text{c}{\text{m}}^{2}\right)} \end{array}$$

To analyze the effects of malnutrition on organs, mice were euthanized (day 16), and the spleen and liver were aseptically removed and weighed on a digital scale. Relative organ weight was calculated as following formula: (total organ weight/body weight) ×100. Other physical parameters were also evaluated, such as the appearance of coat, skin, feces and urine, as well as the behavior of mice.

### Weight-for-Age Relationship (WA)

Anstead et al. (30) described a malnutrition scale mouse model based on the classification by Gómez et al. (31), in which parameters of the animal's nutritional status were evaluated via the weight-for-age relationship (WA). Briefly, the WA of mice was calculated using the following equation: (final weight of malnourished or refed mice/expected weight of malnourished or refed mice based on control mice) ×100.

Analogous to the human classification, in the Anstead model, it is proposed that mice that present WA values between 76 and 90% are classified as having mild malnutrition; those that present WA values between 61 and 75% are classified as having moderate malnutrition; and those that present WA values ≤ 60% are classified as having severe malnutrition.

### Hematological and Biochemical Analyses

On day 16, mice were anesthetized with ketamine (200 mg/kg) and xylazine (16 mg/kg) in a solution that was administered intraperitoneally. Blood was collected (1 ml) by cardiac puncture for hematological and biochemical analyses. The blood was centrifuged at 4000 rpm and the serum was separated. Hematological parameters as well as total blood cell counts were determined. The serum levels of the following biochemical parameters were evaluated: total protein, albumin, glucose, creatinine, creatinine kinase, urea, alkaline phosphatase, alanine aminotransferase (ALT), aspartate aminotransferase (AST), cholesterol, iron, calcium, sodium, and potassium levels. All samples were measured by the clinical analysis platform of Instituto de Ciência e Tecnologia em Biomodelos (ICTB, FIOCRUZ, BR). After blood collection, the mice were euthanized in a CO_2_ chamber.

### Statistical Analysis

The means and standard deviations were determined from at least three independent experiments. Statistical analyses were performed with the program GraphPad Prism 7 (GraphPad Software, USA). ANOVA or *t*-test was applied followed by a Tukey or Mann-Whitney *post-hoc* test, respectively. *P* < 0.05 were considered significant.

## Results

### The Restricted Diet Causes Mild Malnutrition in BALB/c Mice

**Figure 2:**
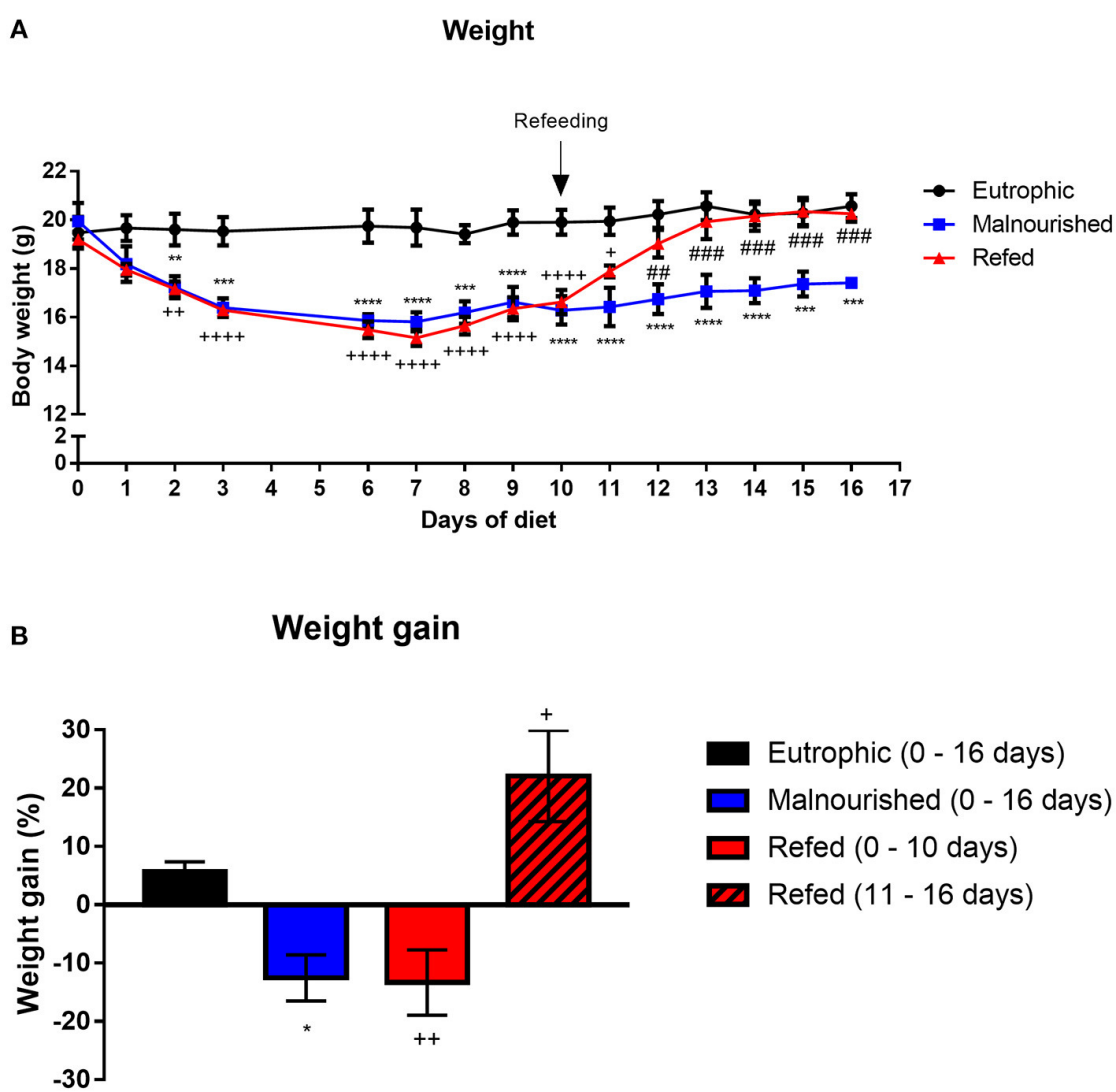
Evaluation of the weight of mice submitted or not to experimental malnutrition and refeeding. Body weight **(A)**. Percentage of weight gained at the end of the experiment **(B)**. At the end of the experiment (day 16), the weight gain percentage was analyzed by dividing the final weight (day 16) by the initial weight (day 0) of the mice. In contrast, for the refed mice, the weight on the last day before refeeding (day 10) was divided by the initial weight (day 0), and the weight immediately before refeeding (day 11) was divided by the final weight (day 16). Black: control group; blue: malnourished group; red: refed group between days 0 and 10; striped red: refed group between days 11 and 16. *p ≤ 0.05; **p ≤ 0.005; ***p ≤ 0.0005; ****p < 0.0001; ^*##*^*p* ≤ 0.005; ^*###*^*p* ≤ 0.0005; ^++^*p* ≤ 0.005; ^++++^*p* ≤ 0.0001. (*) Malnourished vs. control; (+) refed vs. control; (#) refed vs. malnourished. The values are represented by the mean ± standard error of three independent experiments with 15 animals per group.

Mice subjected or not to experimental malnutrition were weighed daily for 16 days. At day 0 (diet introduction), all animal group started with an average weight of 19 g. Mice that received the Restricted diet showed significant weight loss (*p* ≤ 0.009) from day 2 after the introduction of feed, in contrast to control mice that received the Control diet (Figure 2A). Weight loss was observed until the 6th day, and this low weight remained until the end of the experiment in malnourished mice. The refed mice that started receiving the control diet on day 10 demonstrated a significant weight gain immediately 1 day after introduction of the new diet, reaching a weight similar to that of the control mice by the end of the experiment (Figure 2A). Malnourished mice weighed on average 17.4 g at the end of the experiments, while control and refed mice weighed on average 20.5 g. Malnourished and refed (before refeeding) mice lost ~12 and 13% of their body weight, respectively, showing a significant difference compared to control mice (*p* ≤ 0.05 and *p* ≤ 0.009, respectively), which gained ~5% of their body weight. However, after refeeding, refed mice recovered the lost weight, gaining ~22% of their body weight (Figure 2B).

During the experiment, the weight-for-age (WA) was calculated in all groups. One day after the introduction of the Restricted diet, the WA values of the control, malnourished and refed groups were 98.5, 87.9, and 91.3%, respectively. Three days after Restricted diet introduction, malnourished and refed mice presented a reduction in the WA value (80.2 and 82.8%, respectively). At 10 days after Restricted diet introduction, malnourished and refed mice presented WA values of 79.7 and 84.5%, respectively. At the end of the experiments (day 16), malnourished mice demonstrated a WA of 85.2% (Table 2). The reduction in the WA value demonstrated that the Restricted diet led to mild malnutrition. After refeeding, the refed mice reached a WA value similar to that of the control mice (102.9%). Notably, in the control group that received a control diet, the WA values were 97.9, 99.82, and 103.1% on day 3, day 10, and day 16, respectively.

**Table 2:** Weight-for-age relationship (WA) value.

**Groups**	**WA values**			
	**Day 1**	**Day 3**	**Day 10**	**Day 16**
Control	98.50%	97.90%	99.82%	103.10%
Malnourished	87.90%	80.20%	79.70%	85.20%
Refed	91.30%	82.80%	84.50%	102.90%

*Malnutrition scale model in mice based on the classification by Gómez et al. (31). Weight-for-age relationship (WA) classification: a WA value between 76 and 90% classifies mild malnutrition; between 61 and 75% classifies moderate malnutrition; and ≤ 60% classifies severe malnutrition*.

### The Restricted Diet Slows Growth and Causes Body Mass Reduction in Mice

**Figure 3:**
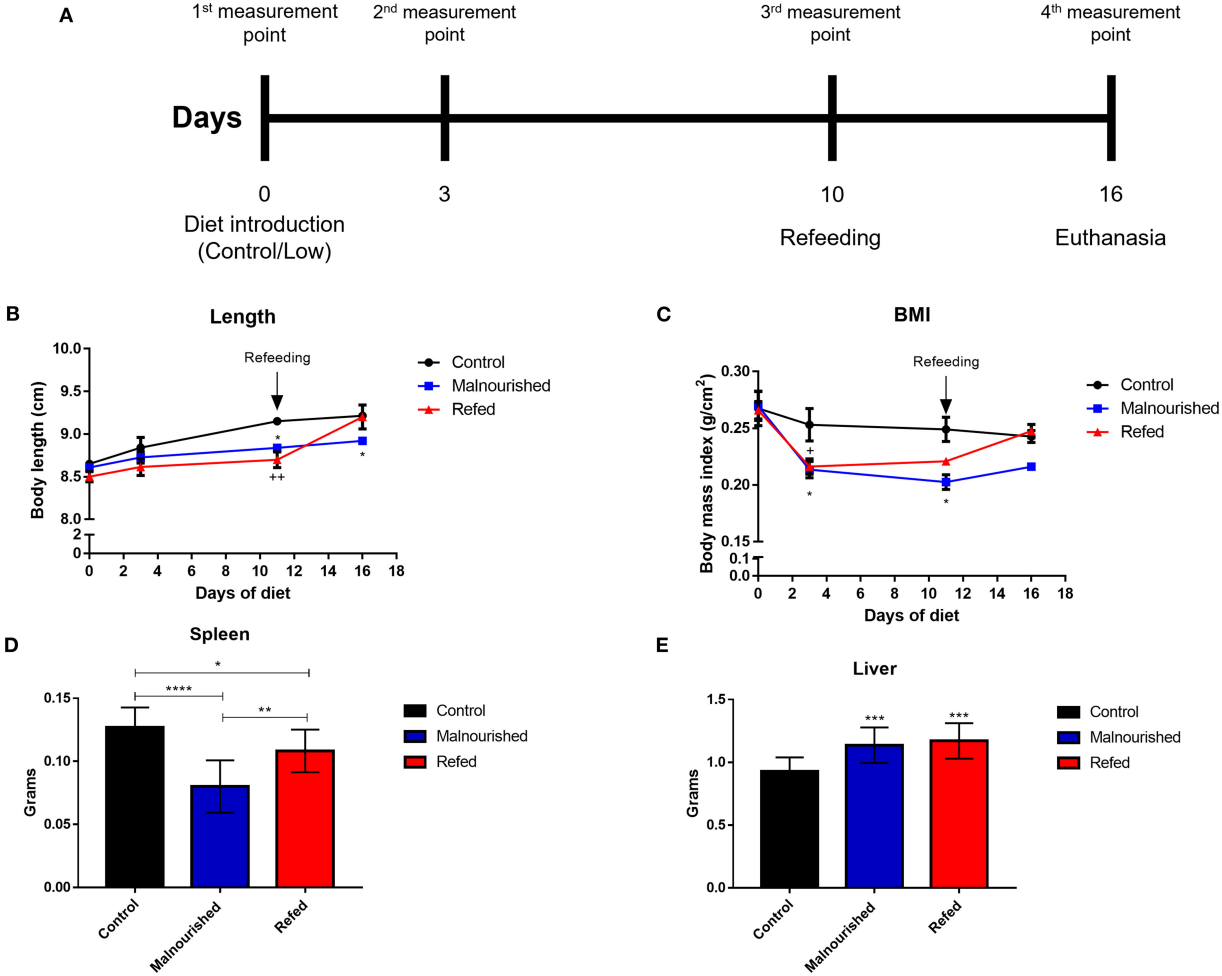
Evaluation of phenotypic markers of mice submitted or not to experimental malnutrition and refeeding. Mice were provided different diets, and body measurements were calculated at days 0, 3, 10, and 16 after diet introduction **(A)**. **(B)** Body length. **(C)** Body mass index. Mice were euthanized (day 16) and total weight of spleen **(D)** and liver **(E)** were weighed on a digital scale. Black: control mice; blue: malnourished mice; red: refed mice. *p ≤ 0.05; **p ≤ 0.005; ***p ≤ 0.0005; ****p < 0.0001; ^+^*p* ≤ 0.05; ^++^*p* ≤ 0.005; (*) Malnourished vs. control; (+) refed vs. control. The values are represented by the mean ± standard error of three independent experiments with 15 animals in each group.

To analyze the nutritional status of the mice by phenotypic markers, the body length of animals was measured on days 0, 3, 10, and 16 after introduction of the restricted diet (Figure 3A). Mice fed the Restricted diet showed slow growth compared to that of control mice (the group that was fed the Control diet), with a significant difference on days 10 and 16 after diet introduction in malnourished mice and on day 10 in refed mice (before refeeding). Refed mice showed a body length increase after the refeeding process, reaching a body length similar to that of control mice (Figure 3B).

Using the body weight and body length, we calculated the body mass index (BMI) of mice fed different diets. Malnourished mice showed a significant reduction in body mass compared to that of control mice. Refed mice showed body mass reduction before refeeding, but after feeding with the control diet, the mice recovered body mass, reaching a value similar to that observed in the control mice (Figure 3C).

The spleen and liver of mice were aseptically removed and weighed to analyze the effects of malnutrition on organ development. Malnourished mice demonstrated a marked reduction in total spleen weight compared to control and refed mice. Refed mice also showed a reduction in total spleen weight compared to the control; however, there was a smaller reduction than that observed in malnutrition mice (Figure 3D). In the liver, both malnourished and refed mice presented a significant increase in this organ compared to control mice (Figure 3E). In addition to the total weight of the organs, the relative weight was also evaluated. In the spleen no significant differences were observed between the groups. In the liver, malnourished, and refed mice showed significative differences (*p* ≤ 0.05 and *p* ≤ 0.009, respectively) compared to control mice (Table 3).

**Table 3:** The relative organ weight of mice submitted or not to restricted diet (on day 16 after diet introduction).

**Organs**	**Control**	**Malnourished**	**Refed**
Spleen	0.62 ± 0.05	0.46 ± 0.10	0.53 ± 0.04
Liver	4.52 ± 0.41	6.51 ± 0.36**	5.85 ± 0.5*

Relative organ weight was calculated as following formula: (total organ weight/body weight) x 100. The values are represented by the mean ± standard error of three independent experiments with 15 animals in each group. *p ≤ 0.05; **p ≤ 0.005. *Statistically significant when compared to the control mice.

During diet maintenance, the physical parameters of the mice were also evaluated. Malnourished and refed mice (before refeeding) showed opaque coats, whitish skin of the paws and tail, and whitish feces. In addition, mice that received the Restricted diet demonstrated agitated behavior, a stooped posture and fed voraciously. All these physical parameter changes were reversed after the refeeding process in refed mice. Three independent experiments were performed, totalizing 15 animals/group. In two of these experiments, one animal from malnourished group died due malnutrition, totaling two animals.

### Malnourished and Refed Mice Showed Changes in Hematological and Biochemical Parameters

To analyze hematological and biochemical parameters, the blood of mice was collected by cardiac puncture, and the serum was separated by centrifugation at 16 days after diet introduction. Regarding hematological parameters, malnourished mice showed a decrease in leukocyte count compared to that of control mice (fed the Control diet), demonstrating a possible influence of malnutrition on the immune system. On the other hand, refed mice showed recovery of leukocyte counts, reaching values close to those of control mice. In addition, other parameters, except mean corpuscular hemoglobin (MCH) and platelets, of refed mice were significantly increased compared to those of malnourished mice (Table 4).

**Table 4:** Hematological parameters at the end of the experiment (on day 16 after diet introduction).

**Groups**	**Control**	**Malnourished**	**Refed**
RBC (mil/mm^3^)	9.20 ± 0.22	9.05 ± 0.21	9.70 ± 0.19^#^
Hemoglobin (g/dL)	13.42 ± 0.33	13.03 ± 0.33	14.11 ± 0.30^#^
Hematocrit (%)	45.52 ± 0.97	42.00 ± 1.75	48.77 ± 0.92^+, ##^
MCV (fm^3^)	49.46 ± 0.65	47.64 ± 5.56	50.19 ± 0.26^##^
MCH (pg)	14.60 ± 0.05	14.25 ± 0.19	14.51 ± 0.12
MCHC (g/dL)	29.50 ± 0.49	29.89 ± 0.29	29.02 ± 0.27^#^
Leukocytes (mil/mm^3^)	4.00 ± 0.35	1.90 ± 0.54*	4.60 ± 0.63^#^
Platelets (mil/mm^3^)	865.8 ± 40.61	974.3 ± 37.52	1,169.0 ± 80.68

On day 16 after diet introduction, BALB/c mice were anesthetized, and blood was collected for hematological analysis. RBC, red blood cells; MCV, mean corpuscular volume; MCH, mean corpuscular hemoglobin; MCHC, mean corpuscular hemoglobin concentration. The values are represented by the mean ± standard error of three independent experiments with 15 animals in each group. *p ≤ 0.05; ^#^p ≤ 0.05; ^##^p ≤ 0.0005; ^+^p ≤ 0.05. (*) Malnourished vs. control; (#) refed vs. malnourished; (+) refed vs. control.

Regarding biochemical parameters, refed mice showed higher glucose levels than control mice, while malnourished mice presented values similar to those of control mice. In addition, malnourished mice showed higher levels of total cholesterol than control mice (Table 5).

**Table 5:** Biochemical parameters at the end of the experiment (on day 16 after diet introduction).

**Groups**	**Control**	**Malnourished**	**Refed**
Sodium (mEq/L)	153.0 ± 1.62	162.0 ± 2.31	154.17 ± 3.05
Potassium (mEq/L)	3.50 ± 0.38	3.31 ± 0.10	3.87 ± 0.45
Glucose (mg/dL)	160.70 ± 16.3	162.90 ± 18.26	245.40 ± 13.56*
Urea (mg/dL)	37.01 ± 14	36.87 ± 2.22	41.02 ± 20.55
Albumin (g/dL)	1.97 ± 0.06	2.16 ± 0.11	2.17 ± 0.14
Calcium (mg/dL)	9.36 ± 0.32	9.89 ± 0.26	9.68 ± 0.30
AST (U/L)	218.70 ± 31.02	106.60 ± 13.64	152.0 ± 2.0
ALT (U/L)	70.0 ± 4.34	47.0 ± 7.80	97.75 ± 4.52
Creatinine kinase (U/L)	380.30 ± 51.49	214.50 ± 41.1	258.30 ± 46.6
Alkaline phosphatase	135.20 ± 5.01	142.0 ± 11.51	145.70 ± 5.49
(U/L)			
Cholesterol (mg/dL)	59.17 ± 4.21	122.20 ± 5.38*	70.67 ± 6.25
Iron (mg/dL)	140.0 ± 14.15	162.80 ± 18.64	149.0 ± 5.20
Total protein (g/dL)	4.47 ± 0.19	4.43 ± 0.10	4.45 ± 0.17
Creatinine (mg/dL)	0.10 ± 0.001	0.10 ± 0.001	0.10 ± 0.001

On day 16 after diet introduction, BALB/c mice were anesthetized, blood was collected, and serum was separated by centrifugation for analysis of biochemical markers. *Statistically significant when compared to the control mice (p ≤ 0.01). The values are represented by the mean ± standard error of three independent experiments with 15 animals in each group.

**Figure 4:**
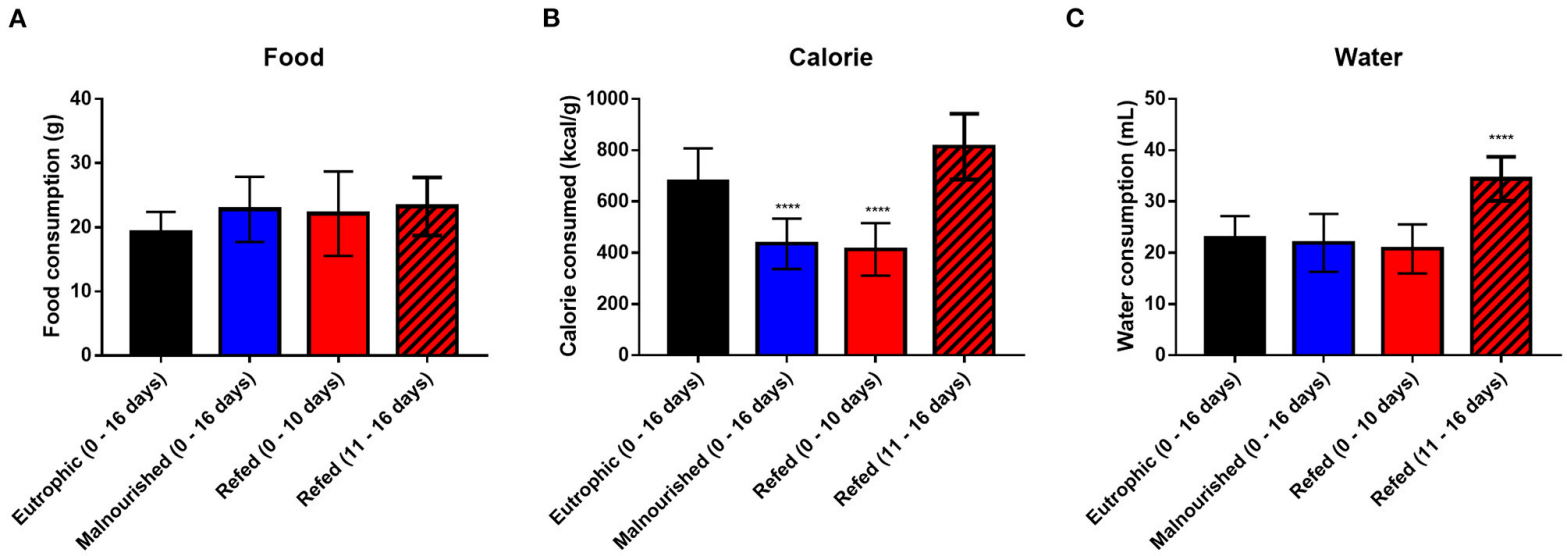
Average consumption of water, food and calories. Daily consumption of food **(A)**; calories **(B)**; and water **(C)**. The values represent measurements from day 0 to day 16 in control and malnourished mice and from day 0 to day 10 and from day 11 to day 16 in refed mice. Black: control group; blue: malnourished group; red: refed group between days 0 and 10; striped red: refed group between days 11 and 16. ****p < 0.0001. (*) Represent differences compared to control mice. The values are represented by the mean ± standard error of three independent experiments with 15 animals in each group.

In addition to body measures, water, food and calorie consumption was evaluated. Malnourished and refed mice (both before and after refeeding) showed no differences in food consumption compared to that of control mice (Figure 4A). However, as expected, malnourished and refed mice (before refeeding) consumed fewer calories than control mice due to a hypocaloric diet (Restricted diet). After the refeeding process, refed mice started to consume more calories, similar to the amount consumed by control mice (Figure 4B).

Regarding water consumption, malnourished and refed mice (before refeeding) showed no difference compared to control mice. However, refed mice (after refeeding) consumed more water than control mice, probably due to the introduction of the normal diet (Figure 4C).

## Discussion

Malnutrition is a serious public health problem that mainly affects people living in precarious conditions. This pathology frequently affects children under 5 years old and is closely associated with infections since it causes immunodeficiency (15). It has been described that the nutritional status of the host directly interferes with the immune system and increases the degree of susceptibility to infectious and parasitic diseases (32). Infections during malnutrition can impair nutrient uptake by the host, leading to worsening malnutrition and generating a vicious circle (20–23).

Malnutrition affects, among other factors, the production of cytokines such as IL-1, IL-12, and IFN-γ, the activity of complement system, phagocytosis, and microbicidal capacity of immune cells (22, 33). González-Martínez et al. (34) suggest that the decreased expression of cytokines related to the Th1 type response (IL-2 and INF-γ) and the increased expression of cytokines involved in the Th2 type response (IL-4 and IL-10) may explain the immune deficiency seen in malnourished children. Immunosuppression, poor sanitary conditions, limited access to health services, exposure to pathogens and the debility that malnutrition causes to the body are risk factors that increase the susceptibility and progression of diseases (20, 35).

To understand how malnutrition impacts disease progression, it is often necessary to use animal models and feeding methodologies that mimic malnutrition. These models are widely used to evaluate the effects of malnutrition on susceptibility to infectious diseases, the immune response and other pathologies related to malnutrition. These factors allow us to analyze the nutritional parameters of the model animals in a controlled way (20, 36). In the literature, many studies use isocaloric diets containing low amounts of protein (37), which can mimic a type of protein malnutrition. However, it is common for children living in precarious conditions to consume diets with both calorie and protein restrictions (38). Therefore, in this study, a feeding protocol using a diet with low protein, lipid and calorie contents was proposed with the intention of mimicking marasmic malnutrition, which is a different approach from those normally used in the literature.

Weanling BALB/c mice were fed the restricted diet and demonstrated marked weight loss 3 days after the introduction of the Restricted diet, and this weight loss remained until the end of the experiment (day 16). This diet was able to induce mild malnutrition in mice, which could be reversed after a refeeding process.

Marasmic malnutrition is an illness that causes many body changes, such as a great loss of weight, muscle mass, and subcutaneous fat, as well as slow growth, lethargy and irritability (8, 10–13). Few studies report the physical aspects of malnutrition in animals during experimental diet consumption, the most common being weight loss monitoring. We, for the first time, evaluated the nutritional status of mice during malnutrition by phenotypic markers. Malnourished mice and refed mice (before refeeding) showed a delay in body growth. However, this delay in body growth was reversed after the refeeding process in refed mice.

One of the parameters used to diagnose malnutrition in adults is BMI, while in children, weight-for-age and height-for-age are the most common (39). However, few studies using these parameters described above have demonstrated a relationship between malnutrition and infectious disease. Here, we demonstrated that the Restricted diet was able to promote a body mass reduction in mice. However, this effect can be reversed after refeeding process. The use of body mass index is more common in studies using rats as animal models (3, 40). Other physical changes were also observed in mice fed the Restricted diet, such as whitish skin and opaque coat, voracious feeding and stooped posture. Hair and skin changes are most common during kwashiorkor malnutrition, in which people present hair and skin depigmentation and skin lesions (6–11). Stooped posture is a clinical feature that has already been demonstrated in baboons with kwashiorkor malnutrition (41). Voracious appetite is a clinical feature often observed in children with marasmus (11, 12).

Marasmus can cause atrophy in vital organs such as the liver, spleen, pancreas and lymphoid tissues (4, 8, 10, 12). To evaluate the effects of malnutrition on internal organs, the liver and spleen of mice were weighed after 16 days of diet introduction. Malnourished mice demonstrated a decrease in spleen weight compared to control mice. Refed mice also showed a decrease in spleen weight, but to a lesser degree than malnourished mice. These data suggest that a short refeeding process reduces, not completely, spleen atrophy caused by malnutrition. Interestingly, in the liver, both malnourished and refed mice demonstrated increased liver weight. Hepatomegaly and fatty liver are common during kwashiorkor (6–8, 10, 11). Nevertheless, it has already been described that children with marasmus also present hepatic steatosis and hepatomegaly (9). It is possible that the livers of malnourished mice contain fat infiltrations; however, more studies are necessary to confirm this hypothesis. Although the present study seeks a methodology that mimics marasmic malnutrition, protein deficiency caused by the Restricted diet (hypoprotein diet) generates clinical features of kwashiorkor, such as skin depigmentation and hepatic alterations. However, we did not detect the presence of edema in malnourished mice, which is the cardinal signal of kwashiorkor (7). Due to the absence of the main clinical feature of kwashiorkor (edema), the Restricted diet can be considered a diet that could mimic marasmic malnutrition.

According to hematological parameters, BALB/c mice fed the Restricted diet (malnourished group) demonstrated an increase in total serum cholesterol levels and a decrease in leukocyte counts, while refed mice showed an increase in leukocyte counts and cholesterol levels similar to those of control mice. However, an increase in serum glucose levels was observed when the mice were refed.

Biochemical parameters are also used in the diagnosis of malnutrition. Albumin, creatinine, total cholesterol, and urea are some of these markers, and the total lymphocyte count is an indicator of inflammation (42–44). Marasmic malnutrition can cause atrophy in lymphoid organs such as the thymus, tonsils and spleen. This process leads to a reduction in leukocyte number (leukopenia), T-cell deficiency, a decrease in neutrophil phagocytosis activity and impairment of antibody formation (4, 12, 21), consequently increasing the chances of developing infections (8). Deposition of fat in the liver in malnourished individuals has been described, causing hepatic steatosis and increasing cholesterol levels (45, 46), a condition that can also be observed during marasmic malnutrition (8, 9). These factors can explain the increase in total serum cholesterol levels and the decrease in leukocyte counts seen in malnourished mice.

The increase in glucose levels seen in refed mice may have been caused by the increase in carbohydrate consumption from the control diet since high consumption of this macronutrient causes this effect (47). In addition, the introduction of a refeeding process after marasmus can cause the development of refeeding syndrome. In this syndrome, an increase in glucagon secretion and a decrease in insulin secretion occur, which cause hyperglycemia and the development of dehydration (4, 48).

Isocaloric experimental diets with protein restriction cause PEM and lead to weight loss in mice. However, many studies have demonstrated that mice increase food intake to try to compensate for the reduced nutrients present in the diet (37, 49–51). We evaluated whether provision of the Restricted diet would result in an increase in the food intake of mice during the malnutrition process. Both malnourished and refed mice (before and after refeeding) showed no differences in food consumption during Restricted diet administration, even though these animals presented voracious appetites. Although refed mice (after refeeding) did not show an increase in food intake, the change in diet (Control diet) allowed an increase in weight gain due to increased macronutrients consumption. Moreover, refed mice increased their water consumption after the refeeding process, which may be related to Control diet consumption and the development of dehydration due to a possible refeeding syndrome (4, 48).

Malnutrition is a severe health condition that can lead to death, mainly when it is associated with an infectious disease. Consequently, a better understanding of the malnutrition/infectious disease relationship is necessary; therefore, the use of animal models and good feed protocols can help us understand the malnutrition/infectious disease relationship.

Taken together, these results indicate that the Restricted diet causes mild malnutrition in mice, leading to bodily changes and some clinical signs similar to those of marasmic malnutrition. In addition, there was no increase in feed intake, which is common during experimental isocaloric protein malnutrition models. For the first time, we demonstrated a protocol using a hypocaloric/hypoprotein/hypolipidic diet that mimics marasmic malnutrition to investigate the relationship between malnutrition and impaired immunity and/or susceptibility to infectious diseases.

## Data Availability Statement

The original contributions presented in the study are included in the article/supplementary material, further inquiries can be directed to the corresponding author/s.

## Ethics Statement

The animal study was reviewed and approved by the Committee on the Ethics of Animal Experiments of the Instituto Oswaldo Cruz (CEUA-IOC, License Number: L-11/2017-A). This study was conducted according to the Guide for the Care and Use of Laboratory Animals of the Brazilian National Council of Animal Experimentation (CONCEA).

## Author Contributions

TF-P and EA-A: conceptualization, investigation, data curation, and writing—review and editing. TF-P, PS-C, and EA-A: methodology and validation. TF-P: formal analysis and writing—original draft preparation. EA-A: supervision. All authors have read and agreed to the published version of the manuscript.

## Funding

This work was supported by Fundação Carlos Chagas Filho de Amparo a pesquisa do Estado do Rio de Janeiro (FAPERJ); Coordenação de Aperfeiçoamento de Pessoal de Nível Superior (CAPES); and Conselho Nacional de Desenvolvimento Científico e Tecnológico (CNPq) and Fundação Oswaldo Cruz (FIOCRUZ). TF-P received a scholarship from CAPES (88882.332475/2019-01) and EA-A was the recipient of a research scholarship from Conselho Nacional de Desenvolvimento Científico e Tecnológico (CNPq). The funders had no role in the study design, data collection and analysis, decision to publish, or preparation of the manuscript.

## Conflict of Interest

The authors declare that the research was conducted in the absence of any commercial or financial relationships that could be construed as a potential conflict of interest.

## Publisher's Note

All claims expressed in this article are solely those of the authors and do not necessarily represent those of their affiliated organizations, or those of the publisher, the editors and the reviewers. Any product that may be evaluated in this article, or claim that may be made by its manufacturer, is not guaranteed or endorsed by the publisher.
